# 10th Anniversary of Cells: Advances in Cellular Immunology—Regulation of Autoimmune Response and Antitumor Reactivity: Are They Two Side of the Same Coin?

**DOI:** 10.3390/cells11244122

**Published:** 2022-12-19

**Authors:** Alessandro Poggi

**Affiliations:** Molecular Oncology and Angiogensesis Unit, IRCCS Ospedale Policlinico San Martino, 16132 Genoa, Italy; alessandro.poggi@hsanmartino.it; Tel.: +39-010-555-8433

## Autoimmunity and Antitumor Response

The innate and adaptive arms of the immune system are involved in maintaining organism homeostasis [1]. Physical, chemical and biological injuries originating internally or externally are sensed by cell subsets of the immune system, and the processes elicited during their recognition aim to restore and maintain tissue homeostasis [1]. However, these processes can be inefficient when it comes to complete and optimal repair, leading to diseases. So, the immune system can be considered as the interface between the self and non-self. Indeed, T cells have been educated to recognize non-self-antigens through complex, and not completely understood, molecular mechanisms that mainly take place within the thymus [2,3]. These mechanisms determine the tolerance to self-antigens and the intolerance to non-self-antigens. Additionally, innate components of the immune system are involved in these processes, as it is well established that professional antigen-presenting cells derive from undifferentiated monocytes that are the main component bridging the two arms of the immune response [4]. Importantly, autoimmune diseases and anti-tumor immunity could represent some similarities, although the former results from an undesired break in tolerance, whereas for the second, the therapist wants to damage the tolerance to unwelcome and harmful self-tumor cells [5]. It is well-established that a key event that drives the immune response is the interaction between antigen-presenting cells (APC) and T cells [3,4]. In this context, the T helper (Th) cells, together with monocyte-derived APC such as dendritic cells (DC), are considered the two key cells that, through several cytokines such as IL1 and IL2, will elicit the immune response [2,3]. The collection of the 10th Anniversary of Cells: Advances in Cellular Immunology is a fruitful series of articles that illustrate some of the hot topics within the field of immune response in autoimmune and neoplastic diseases. Indeed, the relevance of the phenotypic and functional characterization of microglia and macrophages in neuroprotection, especially upon stroke events, can be considered a model for understanding how and when the healing response can be regulated, influencing processes such as angiogenesis and synaptic remodeling that follow brain injury. Indeed, S.R. Var and colleagues have clearly shown the differences between microglia and macrophages, beginning with their classification and pointing out the discrepancies in the literature resulting from the lack of strict specific surface markers. The authors analyzed the respective roles of these two cell populations in acute and chronic stroke, and they consider the therapeutic approaches to polarize and influence the behavior of these cells [6]. On the other hand, the role of specific T-cell subsets such as Th2 T cells is evidenced by their key role, besides the genetic predisposition, alteration in epithelial barrier and immune dysregulation, in the pathophysiology of atopic dermatitis, as detailed by Hidaya A. Kader and co-workers [7]. The role of epithelial barrier is again evident for intestine mucosal cells producing cytokines such as TGF-β, IL-6 and IL-8 instead of TGF-β, IL-33 and IL-25 is a key point to induce, the selection of T cell subsets such as Th1, Th2 and Th17 secreting pro-inflammatory and anti-inflammatory cytokines. This scenario can be reproduced in humanized mouse models of inflammatory bowel diseases (IBD), providing an exceptional tool to study the molecular functions and ontogeny of regulatory T-cells (Treg). Indeed, Sushmita Negi et al. have analyzed the possible specific therapy using humanized mice and Treg [8]. Taken together, these findings pointed out the key role of antigen-presenting cells and T-cell subsets in the regulation and outcome of autoimmune disease. In autoimmune diseases, it is necessary to try to re-establish the lost tolerance to increase the effectiveness the regulatory mechanisms and to impair the anti-self-reaction; however, in neoplasia, it is mandatory to trigger anti-tumor immunity using anti-self-recognition [4,5]. The activation of anti-tumor effector cells should take place within the tumor microenvironment (TME) [4,5]. It is well established that the TME is composed of several cellular and non-cellular components that induce strong downregulation of the immune response [4,5]. Jonathan Anker and colleagues focus on the renal cell carcinomas (RCC) depicting a precise and effective framework of cellular and metabolic targets that should be considered to optimize the effect of immunotherapy. These targets are involved in other neoplasia, and more importantly, the age of diagnosis of several cancers is a key factor that can influence the outcome of the applied therapy. In particular, immunotherapy is influenced by the age of the tumor host [9] and it is evident that most cancers are diagnosed in patients over 60. In this context, Damien Maggiorani and Christian Beauséjour focus on immune response and immune checkpoint inhibitor (ICI) therapy in older patients [10]. This analysis is essentially based on the findings suggesting that immune cell fitness can be impaired in aging and senescence, leading to an inefficient response and clinical outcome [10]. Furthermore, Kumar and colleagues contributed a new approach to improve the efficacy of ICI therapy by co-targeting molecular machineries involved in the modifications of specific RNA to overcome toxicities and drug resistance to antibodies to immune-checkpoint receptors. That epitranscriptomics and general epigenetic molecular mechanisms can play an essential role in determining the cell fate in development and differentiation is becoming increasingly clear [11]. Joyce Taylor-Papadimitriou and Joy M. Burchell have clearly shown the changes occurring in cancer determined by histone methylases and demethylases [12]. These epigenetic regulators can suppress the oncosuppressor genes and activate the oncogenes, leading to cancer progression [12]. It is conceivable that in the near future, the use of specific antagonists will play an additional role together with ICI therapy to trigger or re-wake the anti-tumor immune response. In this context, the correct, simple identification of tumors that show microsatellite instability (MSI) can help us to select the tumors in which an active immune response can be achieved or increased using ICI therapy. James Wei Tatt Toh and co-workers have well characterized a simple, accurate and cost-effective method to test MSI [13]. Importantly, the capillary electrophoresis is associated with computational methods. This new approach uses an automatic computational algorithm to identify the MSI status and the degree of allelic instability.

In conclusion, the collection of the 10th Anniversary of Cells: Advances in Cellular Immunology focuses on some hot topics and novel advancements in our understanding of the mechanisms of regulation of autoimmune response. Indeed, when the cellular processes and molecular mechanisms involved are better clarified, novel designed drugs highly specific for suitable target molecules allow physicians to efficiently treat autoimmune diseases, avoiding the use of unspecific immunosuppressive molecules. On the other hand, preventing the break that leads to autoimmunity will help to reestablish the anti-tumor immune response.

## Data Availability

Not applicable.
